# Induction of homeostatic biological parameters in reward deficiency as a function of an iron-free multi-nutrient complex: Promoting hemoglobinization, aerobic metabolism, viral immuno-competence, and neuroinflammatory regulation

**DOI:** 10.15761/JSIN.1000234

**Published:** 2020-06-29

**Authors:** Kenneth Blum, Bernard W Downs, Manashi Bagchi, Steve Kushner, Bruce S Morrison, Jeffrey Galvin, Kourtney Randsdorp, Justin Randsdorp, Rajendra D Badgaiyan, Eric R. Braverman, Debasis Bagchi

**Affiliations:** 1 Graduate College, Western University, Health Sciences, Pomona, CA, USA; 2 Victory Nutrition International, Inc., Department of R&D, Lederach, PA USA; 3 Dr. Herbs LLC, R&D, Concord, CA USA; 4 ALM R&D, Oldsmar, FL USA; 5 Morrison Family and Sports Medicine, Huntingdon Valley, PA USA; 6 Vitality Medical Wellness Institute, PLLC, Charlotte, NC USA; 7 Functional Freedom LLC, Lancaster, PA, USA; 8 Department of Psychiatry, ICHAN School of Medicine, Mount Sinai, New York, NY, USA; 9 Department of Psychiatry, South Texas Veteran Health Care System, Audie L. Murphy Memorial VA Hospital, San Antonio, TX, USA; 10 Long School of Medicine, University of Texas Medical Center, San Antonio, TX, USA; 11 Path Foundation NY, New York, NY, USA; 12 Department of Pharmacological & Pharmaceutical Sciences, University of Houston college of Pharmacy, Houston, TX, USA

**Keywords:** chronic anemia syndrome, systems biology, reductionist paradigm, VMP35, red blood cells, neutrophils, viral insult, immunity, blood hemoglobinization, aerobic metabolism, anaerobic metabolism, hypoxia, inflammation

## Abstract

**Background:**

A common neurological condition worldwide is Reward Deficiency Syndrome (RDS) leading to both substance and non-substance addictive behaviors, that must be combatted by integrating both central nervous system and peripheral nervous system biological approaches. Integrity of hemoglobin is a crucial determining factor for the overall health functions. Nutrient repletion therapy should be a fundamental strategy to restore the healthy properties of blood. A unique patent-pending iron-free VMP35 formulation was engineered by our laboratory to restore iron-dependent hemoglobin in anemic cells using a proprietary Prodosome^®^ absorption technology. This formulation, containing an array of nano-emulsified botanical ingredients rich in bioflavonoids, strengthens the structural integrity of connective tissues, and potentiates immune competence, cellular aerobic metabolism, and enhances efficient regulation of inflammatory events. We discuss the intricate aspects of strong vs. fragile immunity and consequential inflammatory responses to convey a deeper understanding of the varied and overly complex sequela of immunological behaviors and events. The effect of the VMP35 is mediated through highly absorbable nutritional/nutrigenomic repletion enabling improvements in the systemic set of functional behaviors. In fact, the iron-free VMP35 facilitates a “Systems Biology Approach” which restores hemoglobin status, reverses anaerobic hypoxia, improves competent immune responsivity, and regulates appropriate and controlled activation of general and neuro-inflammatory sequela. Under these pathogenic circumstances, iron-deficiency anemia has been misconceptualized, and a new nosological term, Chronic Anemia Syndrome, is proposed. The comparative therapeutic rationale of Reductionist vs. Systems Biology approaches is also explained in detail.

**Methods:**

The efficacy of the novel therapeutic iron-free VMP35 liquid nutraceutical is detailed in restoring iron-dependent hemoglobin to RBCs and boosting cellular morphology, viability, and immune competence, thereby reducing the need for prolonging inflammatory sequela.

**Results:**

This was demonstrated in a previous IRB approved multi-subject human study. In addition, two recent case studies report dramatic restorative benefits of nutrient repletion therapy of the VMP35 on subjects having experienced near-fatal events, which confirmed the findings explained in this manuscript.

**Conclusions:**

This novel iron-free VMP35 modulates an array of homeostatic biological parameters such as enhanced hemoglobinization, aerobic metabolism, viral immuno-competence, and inflammatory regulation. Further research, examining mechanistic and beneficial effects in athletic performance, is in progress. Importantly, during these troubled immune challenging times, modulating an array of homeostatic immunological and inflammatory dysfunctions are tantamount to improved population outcomes.

**Trial registration:**

The Clinical investigation in a total of 38 subjects was conducted under an Institutional Review Board (IRB) from the Path Foundation in New York, NY (#13-009 April 25, 2013). The two case studies were done at Lancaster General Hospital, Lancaster, PA, and Jefferson University Hospital, Philadelphia, PA, USA. Both studies were retrospectively registered.

## Introduction

A common neurological condition worldwide is Reward Deficiency Syndrome (RDS) leading to both substance and non-substance addictive behaviors, that must be combatted by integrating both central nervous system and peripheral nervous system biological approaches. The authors’ proposal is that the most prevalent form of iron deficiency anemia, including ‘anemia of chronic disease’, is not caused by an iron deficiency [1]. While inflammation and chronic kidney disorders are common symptomatic conditions appearing to result during the anemia, they are a consequence of the etiology of the anemic pathology [2–4]. Low iron and Red Blood Cell (RBC) properties, the evidence of anemia, are the result of a prolific expenditure of alkalinizing histidine molecules from heme protein to maintain blood pH in the critical and optimal life-support zone [1]. The expenditure of histidine, by default, requires the cleavage of iron from heme protein and its repartitioning to other tissue storage depots, i.e. liver, GI tract, bone marrow, brain, etc., ultimately potentiating a dangerous saturation of iron in those tissues with an increase in the ‘overflow’ of unbound iron into the blood known as hemochromatosis, or ‘iron overload anemia’[1]. The hypoxic anaerobic conditions that initiate and ensue are marked by an increase in inflammatory sequela. What has been commonly conceptualized as ‘iron deficiency anemia’ is a deficiency in alkaline buffers. And, since the kidney (tubule) is intricately involved in regulating blood pH via the exchange of ‘OH’ groups off of HCO_3_ (to create CO_2_) and onto CO_2_ to create HCO_3_, i.e. ‘homeostatic counterbalancing’, to maintain pH equilibrium, an increasing anaerobic/hypoxic condition imposes relentless demands on the kidney tubules that prove excessive, exhausting homeostatic maintenance capabilities and leading to kidney dysfunction, disease and ultimately failure. An iron-Free VMP35 Multi-Nutrient Complex has been shown to restore iron-dependent hemoglobin to red blood cells and reanimate neutrophils as well [1]. Moreover, the VMP35 was shown to modulate an array of homeostatic biological parameters: promoting hemoglobinization, aerobic metabolism, viral immuno-competence, and inflammatory regulation [1].

## Reductionist therapy vs. systems biology therapy

There are two types of therapeutic paradigms: 1. Reductionist paradigm, and 2. Systems Biology paradigm [5–8]. A reductionist paradigm is where the intervention is reduced to a single active substance; relies on a single mechanism of action; and reduces the therapeutic target to a singular type of biological molecules, cells, tissues, organs, systems, and/or specific genes, and has the objective of achieving primarily a single beneficial outcome [5,6]. Considering this, in general pharmaceutical interventions are a reductionist paradigm. There are some creative poly-mechanistic exceptions with combined substances like Suboxone and Wellbutrin for example. But even these multi-drug products are employing a targeted mechanistic/pharmacological effect. However, with pharmaceuticals, the outcome can be accompanied by a plethora of undesirable side effects, usually viewed as ‘acceptable risks’(until they are not), from the pharmacological imposition. This and other consequences, like the development of tolerance from feedback signaling and compensatory homeostatic ‘adjustments’ are characteristic of biphasic actions, i.e. the drug exerts its pharmacological effect (phase 1) and the body mounts a retaliatory/adversarial response to the pharmacological effect (phase 2) [5,6]. That effect is a reason that drugs, most obviously observed in neuro-psychiatric disorders and pain medications for example, can appear to become less effective and even experience an escalation in unpleasant side effects in the phase 2 manifestation.

The other paradigm, a ‘systems biology’ therapeutic approach, in contrast to a reductionist approach, is not intended to blunt, block, inhibit, or mask a symptom(s) [7,8]. The systems biology approach relies primarily on therapeutic nutritional strategies to contribute molecular building blocks for the synthesis of the >37 trillion cells that make up tissues, organs, and systems of the body. It is worthwhile to add that systems biology provides the most important criteria for optimal biological functioning via the effective monitoring and interactive feedback of bio-physiochemical signalling/functions of genes, proteins, and their metabolites that assess and regulate metabolic and signalling pathways, which guide biological behaviors [8]. It is important to understand these dynamics in order to design computational models for the elucidation of structure, function, and activity of the molecular determinants. ‘Systems biology’ characterizes the protein-ligand communication on a massive scale. In other words, system functionality requires nourishment and synthesis of molecular components necessary to optimize interactive, interdependent biological functions that positively influence the functional relationships and mechanistic interactions of an entire ‘suite’ (or ‘system’) of biomolecules [7,8]. Rather than being a symptom antagonist, nourishment via the systems biological approach is intended to be a protagonist, restoring and optimizing system functionality at an epigenetic level. It involves optimizing the interconnective signaling of biomolecules downstream, upstream (via feedback) and cross stream (collateral effects) involved in system function [7,8]. This effect is achieved through nutritional/nutrigenomic improvements (i.e. nutrition that influences gene expression, which influences system ‘functional behavior’).

Current therapeutic interventions to reverse, mitigate and manage chronic degenerative disease pathologies, with the goal of relieving suffering and improving the quality of life, primarily utilize reductionist therapies. But, most often, the reductionist therapies have a significant list of potentially serious side effects [6]. Reductionist therapies have had an important role in reducing and managing symptoms of acute crises. However, a review over the last 5 decades would raise questions that even though trillions of dollars have been spent on chronic disease research and therapeutic interventions, have we actually put even a dent in the incidence of chronic degenerative diseases or significantly improved the quality and/or length of life by our strict adherence to a reductionist paradigm? [5–7].

The objective of a systems biological paradigm is to provide nutritional resources that enable the body to create the ideal biological environment to optimize gene expression; enable optimal molecular arrangements and cellular, tissue, organ, and system functions to avail overall health and optimal systemic functionality [7]. A systems biology approach promotes epigenetic corrections and biological recalibration by relying primarily on nutritional strategies to contribute molecular building blocks for synthesis of trillion cells that make up tissues, organs, and systems of the body [6,7].

Based on this premise, our group engineered an SK713 SLP ion-impregnated phospholipid technology (‘Prodosome^®^’) to encapsulate a liquid iron-free phytonutrient, vitamin and mineral VMP35 supplement to facilitate more rapid absorption into the blood. Research from our laboratory demonstrates that iron deficiency anemia (IDA) not caused by nutritional deficiencies, genetic anomalies, or hemorrhages, is caused by a deficiency in alkaline buffers, not iron [1]. This is indeed a novel concept that needs serious consideration from the scientific and health professional community and is an advancement of current therapy. During an anaerobic challenge of hypoxia, iron in the heme protein is cleaved from heme protein, necessary to release histidine, an alkalinizing buffer. The iron is then repartitioned to other tissues such as the liver, lymph, intestine, brain, bone marrow, etc., potentiating oxidative stress and damage in those tissues. Results of clinical research demonstrated that the VMP35 supplement was absorbed and improved hematological, morphological, and rheological properties of the blood in minutes [1,9]. Surprisingly, the iron-free supplement was shown to rapidly restore iron-dependent hemoglobin and reconstitute neutrophils, among other beneficial effects. Results of this research prompted our group to reclassify IDA and anemia of chronic disease (ACD) as Chronic Anemia Syndrome (CAS) [1,9]. The restoration of healthy blood properties should improve overall health. Two case studies are included to confirm this notion.

## Factors influencing blood health

The integrity and property of hemoglobin is an important factor for maintaining good health [9–11]. In other words, if the blood is unhealthy, evidenced by deviations in blood chemistry parameters, whether the individual is symptomatic or asymptomatic, overall health is compromised to some extent [9]. To achieve optimal health, a primary therapeutic objective, in addition to symptom alleviation, is to restore the health of the blood and competent aerobic metabolism [1,9]. Nutrient repletion should be a fundamental strategy to restore healthy hematological, morphological, and rheological properties of blood, including red blood cells and white blood cells (neutrophils). However, commercial agribusiness practices (i.e. chemical fertilizers, pesticides, herbicides, growth enhancers, GMO, irradiation, gassing, coloring, etc.) combined with food processing, functional food property enhancement technologies (taste, texture, etc.), widespread digestive maladies, and the ubiquitous presence of fast food outlets, people are routinely overfed and undernourished [1,9]. What is needed is a supplemental nutrition technology that does not rely on the competence of the digestive system to be absorbed into and benefit the blood [1,9].

To achieve this objective, researchers engineered the VMP35 Multi-Nutrient Complex (MNC) [1,9]. The VMP35 is a patent-pending nano-emulsified iron-free liquid multivitamin, mineral, and phytonutrient complex encapsulated in an SK713 SLP phospholipid envelope (Prodosomes^®^) (Table 1).

The VMP35 is rich in alkalinizing buffers (but is not a ‘high alkaline’ technology) and was shown to restore iron-dependent hemoglobin to red blood cells and reconstitute neutrophils, the most abundant type of white blood cells in the blood [1]. An IRB-approved (need who approved and date) randomized controlled 1-way crossover clinical study demonstrated that blood properties were improved within 5 minutes from intake and sustained for, and even improved at, 30 minutes post intake. A microscopic analysis of various blood properties in 38 male and female individuals demonstrated that the VMP35 provided significant immune supporting and strengthening benefits certainly important for the current cultural immunological challenge [1]. Moreover, the VMP35 is rich in bioflavonoids and phytosaccharides essential for the strength and structural integrity of connective tissues, especially important for promoting immune competence. Collectively, these beneficial effects have been shown to improve cellular oxygen utilization (via hemoglobinization) and aerobic metabolism (which reduces the generation of reactive oxygen species (ROS); strengthen connective tissues, immune competence, and overall health [1,9]. In addition, the benefits include a significant reduction in oxidative stress, cytokine production and the need for chronic inflammatory sequela (appearing to be potent anti-inflammatory effects) [12–16], and, via its improvement in aerobic metabolism, increase protection against opportunistic pathogenic anaerobes (opportunistic in a hypoxic environment) like viruses (e.g. COVID 19), bacteria, fungi etc. [15–19].

A massive upsurge of noxious ROS including small and highly reactive superoxide anion, hydroxyl radicals, singlet oxygen, peroxide radical, hypochlorite radical + hypochlorous acid, as well as other oxidizing agents such as ozone and hydrogen peroxide, also termed ‘redox messengers’, damage and injure biological macromolecules including lipids, proteins, nucleic acids and DNA, as well as trigger enzyme inhibition, all of which potentiate a significant detrimental impact on human health and promote disease outburst [13,14,20–22]. In fact, diverse inflammatory, endothelial, and immune cells generate these ROS by diverse pathways as demonstrated earlier, leading to cellular and tissue damage, onset of an inflammatory cascade and redox signaling events [13,14]. NADPH oxidase is the key enzyme that is intricately involved during this noxious inflammatory cascade. It is important to emphasize ROS production is induced by diverse immune, epithelial, endothelial and dendritic cells, which all simultaneously lead to an inflammatory cascade resulting in chronic inflammation, tissue injury, organ failure and diverse inflammatory disorders [15,16,20–22]. In addition, a cytokine storm, a process whereby white blood cells are stimulated to release inflammatory cytokines, may be triggered, or exacerbated by elevated ROS levels [23–25].

Cell adhesion molecules, including vascular cell adhesion molecule (VCAM-1), intercellular adhesion molecule (ICAM-1), and E-, P- and L-selectins, (basically cell surface lectins that have evolved to mediate the adhesion of white blood cells to endothelial cells and platelets under flow) are upregulated during endothelial pathological activation, further causing inflammatory responses via recruitment, adhesion, and migration of activated leukocytes [16–19]. This accelerates vascular permeability during the pathological activation and thrombosis [17,18]. At this point, it is important to provide a deeper understanding and behavioral biological perspective of inflammation [2,3,22].

## Inflammation and immunity – a deeper understanding of behavioral events

There are several variations on the definition of inflammation. Following are the most common and simplified versions. 1. A localized physical condition in which part of the body becomes reddened, swollen, hot, and often painful, especially as a reaction to injury or infection [24]. 2. Inflammation refers to the body’s process of fighting against things that harm it, such as viral infections, injuries, and toxins, to heal itself [25]. 3. Inflammation is a local response to cellular injury that is marked by capillary dilation, leukocyte infiltration, redness, heat, and pain that serves as a mechanism initiating the elimination of noxious agents, as well as repair and restructuring of damaged tissue [2,22].

Immune responsivity and inflammation comprise a varied and very complex sequela of events [2–4]. The immune system is comprised of the innate and adaptive immune responses, complex mechanistic details of which are beyond the scope of this paper [2,3,24–26]. However, this paper will present the bigger picture of immunological behavior and address the foundational needs for and behavioral aspects of immunological competence and responsivity as it pertains to inflammation.

There are primarily two types of inflammation: 1. Acute inflammation and 2. Chronic inflammation.

1. Acute inflammation is a short-term process occurring in response to tissue injury, usually appearing within minutes or hours. It is characterized by five cardinal signs: pain, redness, immobility (loss of function), swelling and heat [18,23,24, www.nature.com/subjects/acute-inflammation].
2. Chronic inflammation refers to a prolonged inflammatory response that involves a progressive change in the type of cells present at the site of inflammation [23,24]. It is characterized by the simultaneous destruction and repair of the tissue from the inflammatory process, although relentless tissue destruction eventually outpaces tissue repair, which leads to chronic injury and loss of tissue function. It can follow an acute form of inflammation and become a relentless and/or prolonged low-grade form [23,24]. https://www.nature.com/subjects/chronic-inflammation.

The following explains the types and stages of immune responsiveness. In the beginning, immune response consists of recognition of bacterial or viral pathogens by immune cells, which is succeeded either by ingestion, known as phagocytosis or endocytosis, or by activating a signaling cascade that causes production of oxidants to exterminate the noxious pathogens [3,4,25,26]. Subsequently, the infected injured tissue, damaged extracellular matrix and cellular debris are eliminated by the immune cells. Finally, the immune cells repair damaged cells and tissues around the infection [27].

The important element of immune response is the natural killer T (NKT) cell, which exhibits the attributes of both innate and adaptive systems [2,3,28,29]. Innate immunity activates the adaptive system and this communication is bidirectional [28,29]. During the process, these cells act as pattern recognition receptors as well as T-cell receptors and immunoglobulins [28]. Innate immune cells, including dendritic cells, stimulate T-cells through antigens, while adaptive immune cells stimulate innate immune cells through T-helper cell-mediated interferon gamma (IFN-γ which in turn activates the dendritic cell subsets and the macrophages. Innate immunity serves as the first line of defense, while the integral components of adaptive immunity mobilize slowly and consistently [3,4,28,29]. Ultimately, the shared mediators merge innate and adaptive immune function as an integral part of the composite immune system [2,3].

## Determinants of inflammatory responsivity

What is important to understand is that inflammation is a responsive action, not an etiological catalyst. The constitutional strength, form and integrity of the tissues combined with the severity of insult are important factors that determine the extent to which immune system responses are activated and whether inflammation needs to be initiated. The human immune system comprises a wide array of various receptors and signaling mechanisms that recognize and respond to microbial, viral and toxic dangers via signaling cascades that drive inflammation and related processes for direct killing of pathogens [30]. Therefore, inflammation is triggered after numerous preceding immune events are initiated by ischemia and a sudden increase in hypoxicity (a reduction in oxygen availability and utilization) [31]. Hypoxicity begins after either a traumatic or toxicological insult when blood vessels leak transudate (made of water, salt, and protein) causing localized swelling. The burst of reactive oxygen species (ROS) production occurs immediately upon reperfusion of hypoxic cells including antigen-presenting cells like dendritic cells, macrophages, epithelial and endothelial cells as well as neutrophils [32–39].

When the antioxidant defense capabilities of the lung, for example, are overburdened and unable to cope with the increase in ROS, deviations in cellular metabolic function and redox signaling occur. Oxidative stress due to ROS causes proinflammatory cytokine release and enhanced transcription of numerous genes resulting in inflammation, cell injury, and neutrophil recruitment and activation in the lung after ischemic reperfusion (IR) and reoxygenation [40–45]. Cells undergoing reperfusion and reoxygenation following hypoxia produce super oxide radical [46]. In fact, reperfusion of ischemic tissue results in generation of ROS such as superoxide (O2•−), hydrogen peroxide (H_2_O_2_) and the hydroxyl radical (·OH) that leads to an oxidative burst and oxidative damage, such as occurs in the lung tissue [40,46–50]. The release of ROS not only induces cellular lipid membrane peroxidation and the production of inflammatory cytokines resulting in inflammation, but also plays a role in regulating the catalysis of several antioxidant enzymes (e.g., glutathione peroxidase, catalase and superoxide dismutase) as well as key transcription factors such as NF-κB and activator protein-1 (AP-1)[46–48]. However, the more fragile and anaerobic/hypoxic the tissue environment is to begin with, the greater the influx of white cells and thrombocytes will be into those tissues, the more significant the inflammatory response will be, and the greater the magnitude of antioxidant enzymes responsivity will be [46–48]. The quality of all biological functions, including immune regulation, is directly proportional to and dependent upon the constitutional strength and aerobic metabolic capacity of the cells and tissues, and the quality of nutritional resources from which the tissues are made. To that point, innate and adaptive immunity require enough exogenous nutrient resources for proper functioning [43–46].

Chronic inflammatory responses are quite slow and exist for a long time. Long-term inflammation may exist for several months to many years or be a lifelong process in damaged and progressively fragile anaerobic (hypoxic) tissue compartment(s) resulting in additional severe chronic health consequences. In fact, the cause of injury is the guiding factor for chronic inflammation, which largely depends on the type and cause of injury, and its propensity for the body’s ability to repair, heal and overcome the damage [2–4]. Chronic inflammation happens when this response lingers, resulting in a constant state of immunological alert. Prolonged chronic inflammation generally results in connective tissue destruction at a rate that can outpace the repair process. Over time, chronic inflammation can have a destructive impact on your tissues and organs. Some research suggests that chronic inflammation could also play a role in a range of conditions, from cancer to asthma [2–4,51], all of which are characterized by an increased anaerobic/hypoxic tissue environment. Importantly, to the extent that the fragility or frailty of tissues can be significantly strengthened, and normal aerobic metabolism restored, tissue resistance to needing the initiation of inflammatory events can be bolstered [2–4].

## Iron-deficiency anemia (IDA) & anemia of chronic disease (ACD): Misconceptualized

Once inflammation is catalyzed, localized enzymes are activated to control bleeding and prevent infection. Anaerobic/hypoxic conditions of tissues are an antecedent to chronic degenerative disease pathologies [2–4]. Iron-deficiency anemia (IDA) is commonly diagnosed and reported during these pathogenic events, often referred to as anemia of chronic disease (ACD). The notion of our research team is that IDA and ACD have been misconceptualized. Our research demonstrates that the alkalization of the blood requires the expenditure of alkalinizing buffers, such as the release of histidine from iron-bound heme protein. Iron gets cleaved from the heme in order to release the histidine. This process in turn depletes hemoglobin iron, which then appears to be iron-deficiency anemia; but is actually not. Under these pathogenic circumstances, iron-deficiency anemia has been misconceptualized, and a new nosological term, Chronic Anemia Syndrome, is proposed, which more accurately depicts the mechanistic pathology [1]. Restoring oxygen-rich RBC hemoglobin and aerobic metabolism will be crucial to improving immune strength, viral resistance, overall health and reducing the potential induction of inflammatory events. Nutritionally supplying sufficient alkalinizing buffers to pull iron from tissue storage compartments, to which it has been repartitioned, to reconstitute and improve hemoglobin properties will be an important criterion for achieving this paradigm shift [2–4].

Acute inflammation, the short-term response, occurs due to tissue injury from trauma or toxin exposure/infection, and is an inflammatory response that appears within minutes or hours of the insult [2–4]. Acute inflammation exhibits primarily five characteristic signs including pain, swelling, heat, redness and loss of function or immobility. Inflammation is intended to be a natural down-stream part of the healing process and only becomes problematic if prolonged and/or excessive [2–4]. As indicated, the duration of inflammatory events is inversely related to the constitutional strength and oxygenating potential of the afflicted tissue and/or the duration and intensity of the injurious source/cause, such as an infectious virus, bacteria, etc. Moreover, an increased anaerobic environment (hypoxic state) increases the duration and intensity of the inflammatory process. The interactions between chronic inflammation, endothelial dysfunction, and oxidative stress have been studied extensively [2–4]. However, numerous unfavorable antioxidant therapy trials are rife with apparent contradictions to popular supplemental recommendations. A greater understanding of the etiology of oxidative stress remains to be fully understood and explored. The terms hypoxia, anaerobic and acidic are synonymous indicating an inability to effectively use oxygen in cells; i.e. oxygen deprivation as opposed to oxygen deficiency. The question is what happens to the oxygen we are breathing when it is unable to be effectively utilized for aerobic glycolysis, for example [1]. The answer is that oxygen that is not able to be effectively managed in cellular metabolic events, instead oxidizes cell membranes, lipids, cross-links proteins, and damages DNA among other consequences. Supplemental oxygen therapy in these pathogenic situations can significantly increase oxidative stress, damage, and cell destruction.

Metabolic dysfunction caused by oxidative damage is therefore not primarily caused by a deficiency of antioxidant supplements. A deficiency or impairment of the functionality of intracellular organelle machinery is a greater contributing factor. While supplemental antioxidant consumption may provide some advantage in the treatment of chronic oxidative stress, a preponderance of scientific evidence is still lacking tangible benefits in the treatment of acute and dangerous conditions including ischemic-reperfusion injury, adult respiratory distress syndrome (ARDS), sepsis, multiple forms of cancer and all other anaerobic pathologies [52]. In contrast, hypoxia and anaerobic glycolysis are antecedents to inflammatory events and chronic degenerative disorders [12]. Restoration of aerobic metabolism is essential to restoring healthy metabolic functions.

Up to this point we have discussed various conditions that trigger immune responsivity and promote acute and chronic inflammatory sequela. Conventional medical interventions are tasked with reducing the symptoms of inflammation, relieving pain, swelling, edema, etc., and relieving suffering; a worthy and laudable objective [2–4]. The symptomatology and diagnostic assessments of the various conditions generally indicate the options for pharmaceutical interventions. However, improving the constitutional strength of biological tissues, in addition or in contrast to managing symptoms to reduce suffering, is a foundational therapy crucial to promoting and maintaining overall health and improving immune responsivity. Different therapeutic interventions have different mechanistic actions with significantly different objectives, especially regarding the biological management of inflammatory events, which has been addressed above. Restoring healthy aerobic metabolism must first begin by improving the health of the blood.

## Restoring hemoglobin, reducing hypoxia, boosting immune competence and managing inflammatory catalysis – A systems biology approach

### The catastrophic consequences of misdiagnosis:

Misdiagnosis and mistreatment of disease conditions is a serious infraction. In this paper, we describe the diagnostic misunderstanding of iron deficiency anemia (IDA) and the consequences of mistakenly prescribing excessive iron supplementation when an anerobic/hypoxic state forces cleaved iron from hemoglobin (to release alkalinizing histidine) to be repartitioned to other storage depots inducing ‘tissue-iron-overload’ up to and including severe toxicity, as in hemochromatosis. These pathogenic events are more accurately termed ‘Chronic Anemia Syndrome’ (CAS) as iron is still in the body; just not within RBCs as heme has been dismantled or deconjugated to release alkalinizing histidine.

As our group has previously published, optimal health requires an optimal ability to effectively utilize oxygen and water [1,9], not just forcing an extraordinary amount of oxygen into the tissues (via ventilator therapy), which can (and does) induce severe oxidative damage when the intracellular machinery to enable its effective use is lacking. Dr. Eddy Fan, an expert on respiratory treatment at Toronto General Hospital, stated, “One of the most important findings in the last few decades is that medical ventilation can worsen lung injury - so we have to be careful how we use it” [53–58].

People especially vulnerable to severe infections and sepsis are the elderly and those with one or more chronic co-morbidities, which are already anaerobic pathologies. This is especially notable with people presenting with a high viral titer count. For this reason, the use of ventilator therapies may increase oxidative damage and destruction to vital tissues [56].

Giannini *et al.* recommended the use of resuscitation therapy for venous micro-thrombosis and in similar cases of pulmonary complications known as venous thromboembolism (VTE) [56]. VTE is a condition where blood clots are formed in the deep veins of the leg, groin or arm, a condition well-known as disseminated intravascular coagulation (or thrombosis) or deep vein thrombosis (DVT), which subsequently progresses in blood circulation leading to pulmonary embolism. Dr. Giannini indicated that resuscitations and intubations in ventilating lungs are a completely wrong approach to eradicate certain viral infections [56]. This is contrasted with current medical and scientific literature, specifically research coming out of China, which until mid-March, claimed that anti-inflammatories should not be used [58]. Giannini stated, “Here the inflammation destroyed everything and prepared the ground for the formation of thrombi”.

The current evidence appears to indicate that the etiological mechanism initiating the pathophysiological events begins by impairing the oxygen-carrying properties of the blood, inducing an anaerobic/hypoxic state that promotes inflammatory sequela and potentiates the formation of thrombi; and not the reverse [58]. As such, these clinical observations confirm that simultaneous with the inflammatory response in an anerobic/hypoxic condition in the blood, more red blood cells and platelets would, in a defensive response, be produced that could lead to intravascular coagulation or thrombosis [58].

The proposition of our group is that the induction of an anaerobic/hypoxic state is the antecedent to pathological pulmonary, renal, and cardio-vascular events that after progressing further, induce the formation of blood clots and organ distress, which has been observed. The clinical findings of Giannini *et al.* confirm our earlier findings that Chronic Anemia Syndrome impairs the oxygen carrying properties of the blood. This impairment exacerbates and is an antecedent to most, if not all, viral, bacterial/infectious, and chronic degenerative disorders [1,9].

## Disease pathophysiology

A compromised immune system, infections, anemia, and an array of inflammatory events are evident in diverse disease pathologies in humans and animals [9,10]. As already mentioned, these noxious events are preceded and characterized by an increase in anaerobic metabolic events [9–11,59–63]. Optimal health is the result of the body’s ability to successfully maintain the most ideal biological environment for optimal gene expression and cellular functioning. Aerobic cellular events are important for human life, optimal gene expression, and healthy mental and physical performance. An important property of maintaining the ideal biological environment is pH homeostasis. A highly efficient pH buffering system, for example maintaining a blood pH between 7.35 and 7.45, is required for maintaining optimal and usable oxygenation of the blood in addition to many other biological processes [9–11,59–65]. Optimizing the ideal pH in the blood is the result of the compensatory homeostatic exchange of acid and alkaline pH buffers. A pH of the blood below 7.35 is acidemia, while a pH above 7.45 is alkalemia, with a pH of 7.40 being ideal [59–62,64–68]. Due to the importance of sustaining a pH level in the narrow specified range, the human body exerts a compensatory mechanistic acid/alkaline exchange via Homeostatic Counterbalancing [64–68].

As indicated earlier, increased cytokine production, inflammatory responses and compromised immune health are characteristic of chronic diseases, which exhibit an increased inability to effectively utilize oxygen, resulting in increased hypoxia, anaerobic metabolic events, and lactate accumulation in the body. Impairment of oxidative pathways induces significant lactate production via anaerobic glycolysis, resulting in a net gain of H+ (i.e. protons) with increasing cellular acid burden, thereby decreasing the blood pH, described as an increasing anerobic or hypoxic condition [64–67].

A progressive inability of cells to effectively use cellular oxygen leads to Progressive Acidemia, a metabolic shift toward cellular anaerobic glycolysis, and a compensatory expenditure of alkalinizing histidine molecules from the heme protein of deconjugated hemoglobin, which releases iron. Iron is taken out of circulation and accumulates in the liver, bone marrow, and other organs, which appears to be iron deficient anemia (‘IDA’) but can result in dangerous accumulations of iron in the various tissue storage depots [9,10,67,68]. Our group suggests that ACD and IDA have been misconceptualized and asserts that Chronic Anemia Syndrome (CAS) is a more appropriate and accurate descriptor for the anaerobic/hypoxic conditions inducing chronic disease pathologies.

In addition to iron accumulating in certain organs, the consequences of an increasing anaerobic/acidic environment, especially in the blood, can manifest in a number of ways, in various tissues, and produce a wide range of symptoms and pathological manifestations including chronic and acute infections, flukes, vaso-occlusive incidences, CVD, hypoxia, strokes, kidney diseases, cancers, diabetes, tuberculosis, HIV, endocarditis, osteomyelitis, inflammatory bowel diseases such as Crohn’s disease, etc. [9–11,67,68]; and oxygen deprivation-exacerbated reward deficiency syndrome-disorders (RDS) in which functional inter-connectivity (i.e. cross talk) and neuroplasticity of brain cells are impaired; among other consequences. This type of impairment can exacerbate and/or lead to excessive reward-seeking thoughts and behavioral disorders such as ADD, ADHD, Obstinate Defiance Disorder (ODD), hypersexuality, substance use disorder (SUD), dementia, tics, Tourette’s, Parkinson’s Disease, sleep disorders and vivid nightmares, depression; obsessive, compulsive, impulsive and addictive behaviors; autism spectrum disorder, intermittent explosive disorder, bipolar disorder, uncontrolled cravings, extreme self-medicating behaviors, stress intolerance, fatigue, relapse, and poor decision making, among many others types of RDS behaviors [9,10,67,68].

An important strategy is to restore oxygen utilization for normoxic aerobic (alkaline) metabolism and to restore blood oxygen level dependent signaling and functioning to optimize gene expression, neurotransmitter cross-talk in the brain reward cascade (BRC) for optimal dopamine metabolism, and reward processing [69].

## Methods

### Engineering of VMP35 supplement manufacturing

A suite of carefully selected and science-based micronutrients including vitamins, minerals and botanical phytonutrients was manufactured into a finished nutraceutical using a state-of-the-art novel proprietary SK713 SLP multi-lamellar clustoidal non-GMO phospholipid nutrient absorption/delivery technology (Prodosome^®^) (Table 1). The manufacturing technology takes place in a proprietary multi-step cGMP and NSF-certified manufacturing facility. The resulting nutraceutical technology is biodegradable and biocompatible.

Following is an overly simplified process description:

- In the first step, a specific amount of sterile reverse osmosis water is placed into a dynamic vortex spinning at very high speeds for a specified amount of time to produce structured water.
- In the second step, the manufacturing of SK713 SLP is performed using a non-GMO lecithin containing a minimum of 85% phosphatidylcholine (PC) which is impregnated and saturated using solar-dried electrolytes to ensure the availability of free ions, which will amplify the ionic properties of the multi-lamellar clustoidal phospholipid spheres. The PC is then slowly and carefully added to the dynamic vortex of water.
- In the third step, a proprietary combination of research-driven struc-turally diverse antioxidants, multivitamins, micronutrients, minerals, and standardized botanical phytonutrients are carefully, sequentially, and progressively introduced and thoroughly blended in a high-shear wet milling treatment to create a nano-emulsion.
- The final step involves the extensive blending of the nano-emulsion and ion-enhanced PC to achieve the Prodosomed encapsulation technology, which produces this patent-pending multi-lamellar energetically enhanced clustoidal ‘Prodosomal’ liposome-type encapsulated VMP35 formulation.

### A nutraceutical “systems biology” therapeutic approach: Revelations of a clinical approach

#### Study design:

A Clinical investigation was conducted in a total of 10-male and 28-female subjects (age = 22–82 years old) who were recruited from medical health clinics during interviews in Woodbridge and Perth, ON, Canada. Institutional review board (IRB) approval from the Path Foundation in New York, NY (#13-009 April 25, 2013) was obtained. All subjects signed an informed consent form.

This controlled one-way crossover study evaluated the efficacy of VMP35 supplementation on blood oxygenation and hydration, and compared to the control group, at baseline, 5 min and 30 min post-treatment, respectively. Alterations in peripheral blood smears (PBSs) from baseline (0 min) were assessed using live blood cell imaging (LBCI) with phase contrast microscopy at 5 min post-control intake, as well as 5- and 30 min post-VMP35 supplementation. Live blood cell imaging conducted by Veritas Health Inc (Woodbridge, ON, Canada) using an Olympus BX-30 light microscope. PBSs were evaluated in both placebo and VMP35 supplemented subjects during a period of 0, 5, and 30 min post-treatment. Adverse event monitoring was strictly enforced.

### Cell imaging

Live blood cell imaging was performed by Veritas Health Inc (Woodbridge, ON, Canada) by using an Olympus BX-30 light microscope integrated with a 150-W lightbox with fiber-optic cable assembly. Optical resolution exhibited a high level of cell definition, morphology, and clear distinction of the cell membranes using lens configuration of 10X and 100 X to achieve about 1,000 X magnification. Especially, oil immersion integrated finer resolution and brightness.

### Blood smear procedure

peripheral blood smears (PBSs) were produced using a Bayer Single-Let Disposable Lancet 23G 2.25 mm sterile single-use lancing device (Whippany, NJ) from the fingertip. PBSs were drawn from all subjects over a period of 0, 5, and 30 min post-treatment. An exceedingly small amount of capillary blood was transferred directly on pre-cleaned microscope slides. The slide was then transferred directly to the microscope for viewing. Identical process handling procedures were conducted to avoid artifacts.

### Case studies

Two Case Studies were Conducted to Determine the Effects of Iron-Free VMP35 Supplementation in Subjects with Serious Health Crises

Both case studies were conducted under the strict supervision of physicians. In these case studies, each patient signed an approved consent form and provided thorough hospital records of pertinent medical information obtained along with physician and therapists interaction with the research team. Both studies were conducted under the strict supervision of physicians.

### Case study #1

Justin and Kourtney Randsdorp, Certified Occupational Therapists, provided Information for DH on Apr 30, 2020, with permission from DH and the Attending Physician’s Approval

Medical History: A 63-year-old male subject, a non-smoker, with a previous medical history indicating a significant T4aN0 adenocarcinoma on his sigmoid colon; status post (S/P) lower lobe lung resection on 2^★^ coronary artery (CA) with METS from colon. Subject indicated with all activities of daily life scale (ADLs)/ Instrumental activities of daily living scale (IADLs) prior to surgery, reports of some right knee pain and general joint pain, brain fog, and reduced energy. During follow up surveillance imaging on April 8, 2020, it was detected that the subject had a right lower lobe lung cancerous nodule, which initially decreased in size and was observed to be presently growing again, while the occurrence of new enlarging left lower lobe lung cancerous nodule was reported on Apr 8, 2020. Surgery was scheduled for 6 weeks later (which was rescheduled and performed a week later). Active problems included (i) lung nodule, (ii) cancer of sigmoid colon (CMS/HCC), (iii) colonic mass, (iv) abnormal EKG, (v) S/P angioplasty with stent, (vi) coronary artery disease, (vii) nonalcoholic steatohepatitis and (viii) elevated cholesterol, (ix) pain of meniscus of left knee, and (x) torn rotator cuff. Subject has no known allergies. No negative findings were observed in radiology and cardiology examination.

In addition to the following oral medications, aspirin (Ecotrin 81 mg/day), atorvastatin (Lipitor, 40 mg/day at bedtime), vitamin D3 (cholecalciferol) 4,000 IU/day, Fluzone Quadrivalent 0.5 ml SUSY (once to be administered by the pharmacist for immunization), nitroglycerin (NITROSTAT, 0.4 mg Sublingual ab, place 1 tablet under the tongue every 5 min as needed for chest pain), Probiotic (1 capsule/day) and therapeutic multivitamin minerals tablet (Theragran-M tablet, 1 tablet/day), Subject started taking the VMP35 MNC (Prodovite^®^) and discontinued the Theragran-M tablet approximately 2 weeks later in March.

During examination on Apr 15, 2020, diagnostic analyses confirmed previous assessment and indicated that active problems included (i) lung nodule, (ii) cancer of sigmoid colon (CMS/HCC), (iii) colonic mass, (iv) abnormal EKG, (v) Stent Placement angioplasty, (vi) coronary artery disease, (vii) nonalcoholic steatohepatitis, and (viii) elevated cholesterol. During examination of chest/pulmonary tissues, subject had normal breathing and no sound or respiratory distress, no wheezes, and no rales. In abdominal examination, bowel sounds were normal. No distension, no tenderness, and no rebound, while in musculoskeletal examination the subject had normal range of motion, no edema, tenderness, or deformity. In neurological examination, the subject was reported to be smart and oriented to person, place, and time. No cranial nerve deficit and normal coordination. Skin was warm and dry, and no rashes. Subject is not diaphoretic, and no signs of erythema or pallor. In psychiatric evaluation, the subject was reported to have normal mood, normal behavior and affect, as well as judgement and thought content were normal. In constitutional evaluation, the subject was reported to be well-developed, well-nourished and no signs of distress. No negative findings were observed in radiology and cardiology examination. Preoperative diagnosis showed the presence of left lower lobe lung cancerous nodule. Surgery was performed on April 15, 2020, using LEFT robotic assisted thoracoscopic surgery, and left lower lobe wedge resection. Postoperative diagnosis also indicated likely left lower lung lobe consistent with colon metastasis.

During the robotic surgery, no evidence of metastatic disease was noted on entry into the chest. Furthermore, along the posterior-lateral aspect of the lung no nodule was identified. Frozen section analysis confirmed the presence of malignancy and a negative staple line. Hemostasis was noted along the staple line.

In addition to existing prescribed medications stated above, Subject started taking 4 oz VMP35/day from March 1, 2020 and increased the daily dose to 6 oz/day from the 3rd week. On May 1, Occupational Therapists attending to DH reported that after regularly consuming the VMP35 over a period of 60 consecutive days, the reported observations were recorded in both pre- and post-operative conditions.

### Case study #2

#### History:

On Oct 10, 2018, a 33-year old male subject (GLK) was involved in a motorcycle accident with extensive life-threatening injuries, including extensive fractures of the left ribcage, pelvis and femur, and was immediately admitted to Paoli Hospital (Paoli, PA). Upon making the Subject sufficiently stable, 9 hours later Subject was air-transferred to Jefferson Hospital (MRN 402092750 and 2065716), Philadelphia, PA. The Subject evidenced severe weakness and was diagnosed with numerous severe fractures and problems including (i) severed femoral artery, (ii) ruptured spleen, (iii) bleeding in the space between lungs and chest wall, (iv) fracture of the left acetabulum, (v) flail chest and (vi) collapsed lung.

Due to the life-threatening severity of injuries, Subject’s health was too fragile for attending surgeons to perform necessary reconstructive surgeries upon entry to the hospital. Surgical intervention was questionable in the first 3 days. Examination exhibited significant blood loss and anemia. Table 2 exhibits time-dependent clinical parameters including hematocrit level, hemoglobin level, platelet count, red-blood and while blood cell counts. On the very same day, within 6 hours of the accident, a complete blood transfusion of 1.5 times body capacity, was given due to excessive bleeding with an additional 5-pint plasma infusion. Family members were notified of the severity of GLK’s injuries and advised to get to the hospital ICU as soon as possible. Within 3 days of the accident (on Oct 13, 2018), the subject started consuming a daily dose of 6 ounces of the iron-free VMP35. Within 3 hours after consuming the VMP35, subject’s hemoglobin was normalizing.

Two days later, by Monday, October 15, 2018, Subject had become sufficiently strong enough for surgeons to perform extensive reconstructive surgical procedures and Subject was able to receive prescribed medications as per the schedule. Subject’s recovery far exceeded projected expectations in regard to the duration of incapacitation and improvements in quality of life. Subject was released from hospital on Oct 25, 2018. The subject continued consuming 4 ounces of VMP35 from Oct 25, 2018, until Jan 2019, whereupon he continued with a maintenance dose of 2 ounces per day.

Subject underwent extensive daily physical therapy. The physical therapist projected subject’s inability to walk without assistance until the Summer of 2019, and possibly resume his daily work and a more active lifestyle from late Spring of 2019. However, the subject started walking without any additional assistance of crutches or a cane, from Dec 18, 2018, significantly ahead of projections. The Subject stopped taking prescribed/OTC pain medication on December 6th. Subject was able to engage in rigorous physical winter sports activities (i.e. ice hockey) in March of 2019, well ahead of the projected summer schedule.

## Results

### VMP35 clinical study

The study was conducted in a total of 38 subjects (age = 22–82 years). VMP35 induced the restoration of hemoglobin and neutrophils leading to dramatic improvements in morphological, hematological, and rheological factors in the blood following 5 min of administration and a sustained effect was observed for at least 30 min post supplement intake. Improved blood rheology was observed by videographic recording of live RBC movement on microscope slide, which exhibited the improvement in blood rheology. The still photographs demonstrate a significant reduction in RBC aggregation, improved RBC morphology, and distribution. The VMP35 induced rapid improvements in blood properties, restored RBC Hb saturation and morphology, and improved neutrophil morphology within 5 min that were sustained for 30 min post-supplement intake. Moreover, prompt, sustained, and progressive results were observed in the treatment groups

Our group demonstrated that highly bioavailable and comprehensive supplemental nutrient repletion from the iron-free VMP35 provides the buffers necessary to halt iron-dependent heme expenditure from hemoglobin and reconstitute RBC hemoglobin [1]. Excess accumulated iron is therefore effectively pulled from other tissue storage depots, enabling rapid re-constitution of red blood cell hemoglobin, improving oxygen-carrying ability and neutrophil morphology [1,9]. Contrary to conventional perspectives, this research revealed that non-genetic, non-hemorrhagic and non-nutritional deficient iron-deficiency anemia (IDA) is caused by a deficiency in alkaline buffers instead of an iron deficiency. As such, the wisdom of iron-repletion therapy, which can temporarily raise blood-iron levels, is probably contraindicated and should be carefully reconsidered.

### Case studies

Results of Two Case Studies to Assess the Effects of Iron-Free VMP35 Supplementation in Subjects with Serious Health Crises. As indicated earlier, both studies were conducted under the strict supervision of physicians.

### Case study #1

Justin and Kourtney Randsdorp, Certified Occupational Therapists, provided Information for DH on Apr 30, 2020, with permission from DH and the Attending Physician’s Approval.

Prior to lung resection, subject started taking VMP35 with continued exercise routine of running 3 miles, 3-times per week. Throughout 60-day VMP35 implementation, the following benefits were noted:

1. Improved stamina while running (increase from 3 miles to 3.5 miles with perceived rate of exertion decreasing with increased mileage)
2. Less knee and joint pain with activity. Almost no pain while running.
3. More clearer thinking and focus
4. Increased energy

After surgical procedure, subject reported a “quick and consistent” recovery from lung surgery. Objectively, subject’s ability to walk 2.5 miles without pain within two weeks of surgery, increased to walking 3+ miles within 3 weeks of surgery without any perceived pain. Subject indicated no shortness of breath or need for energy conservation training during daily activities or exercise routine. Oxygen Saturation remains greater than 97% with exertion. Recommendation for home exercise program (HEP) to continue upgrading the aerobic exercise routine and progress to heavy IADLs as tolerated. Subject reported to continue supplementation of VMP35 due to subjective and objective improvements observed during both pre- and post-surgery.

### Case study #2

**Figure 1.**
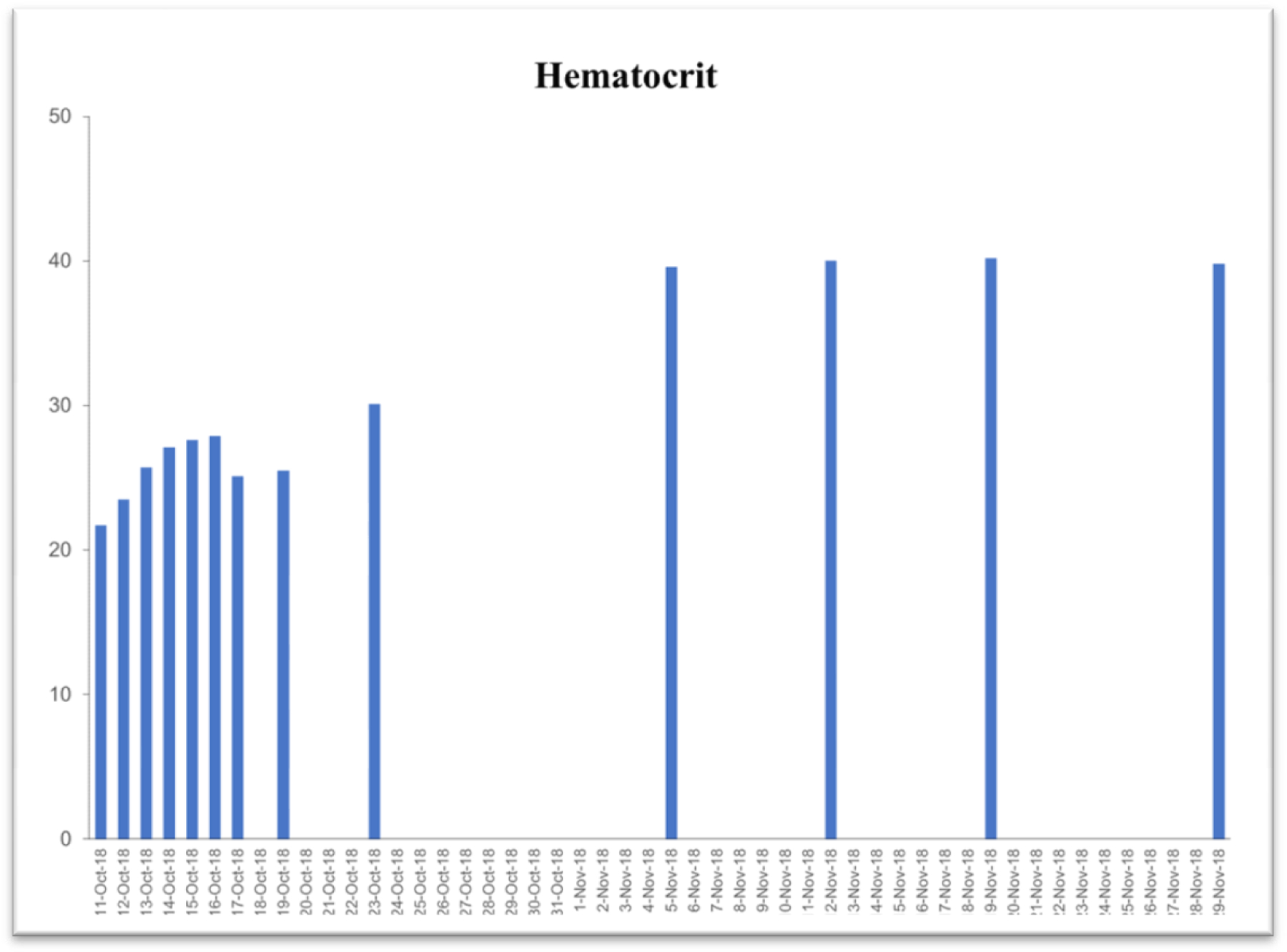
Graph represent hematocrit

**Figure 2.**
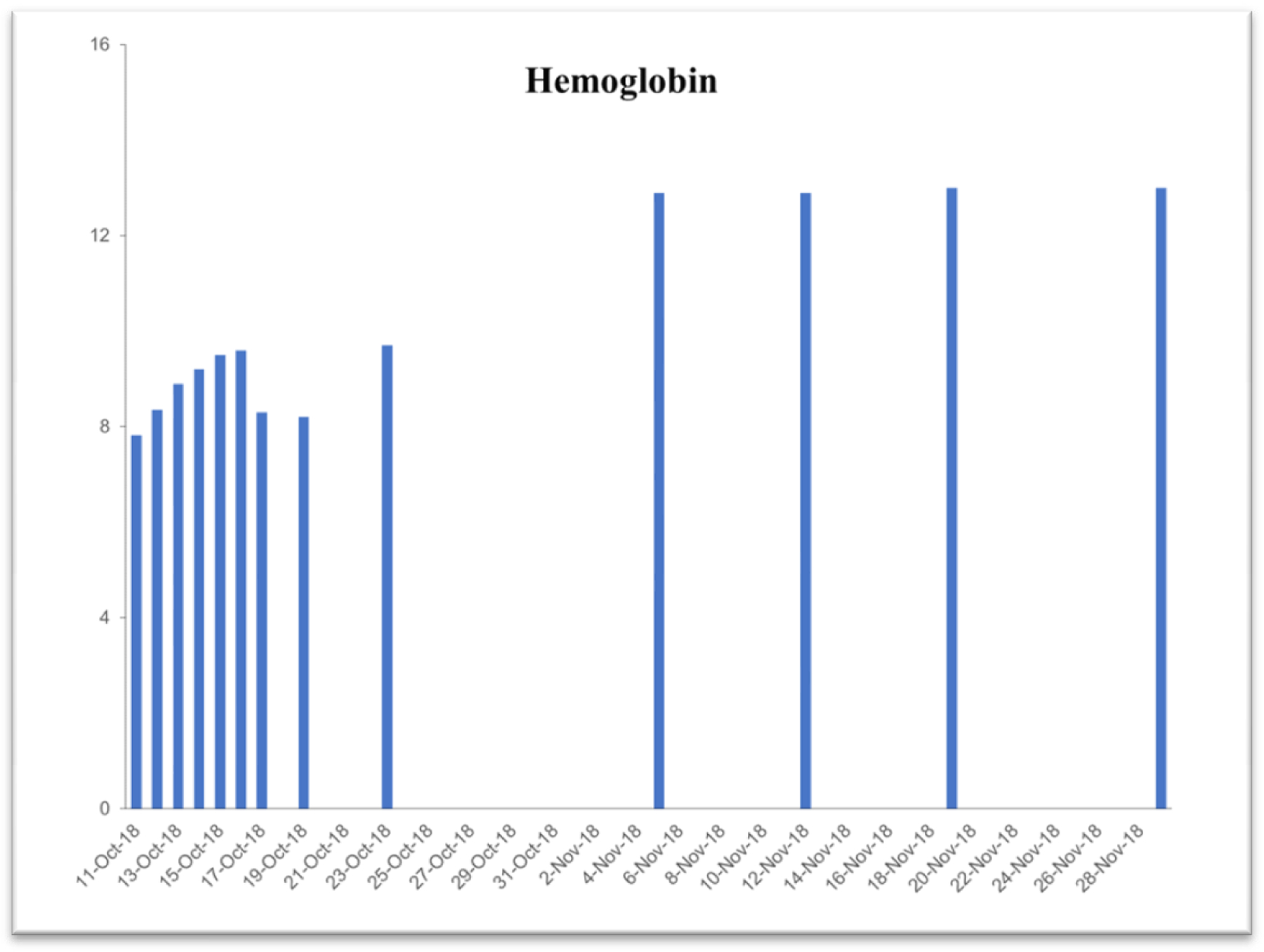
Graph represent Hemoglobin

**Figure 3.**
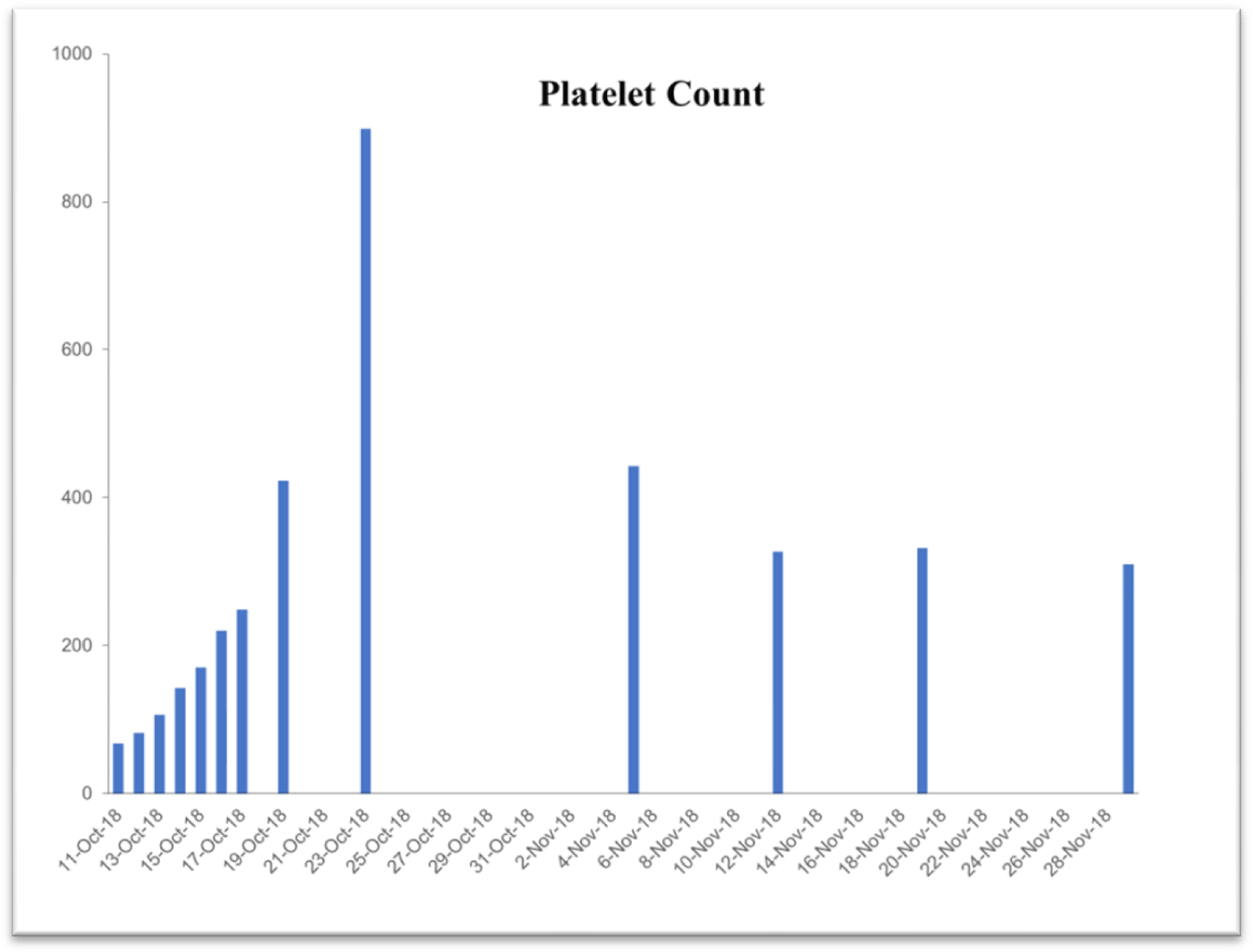
Graph represent platelet count

**Figure 4.**
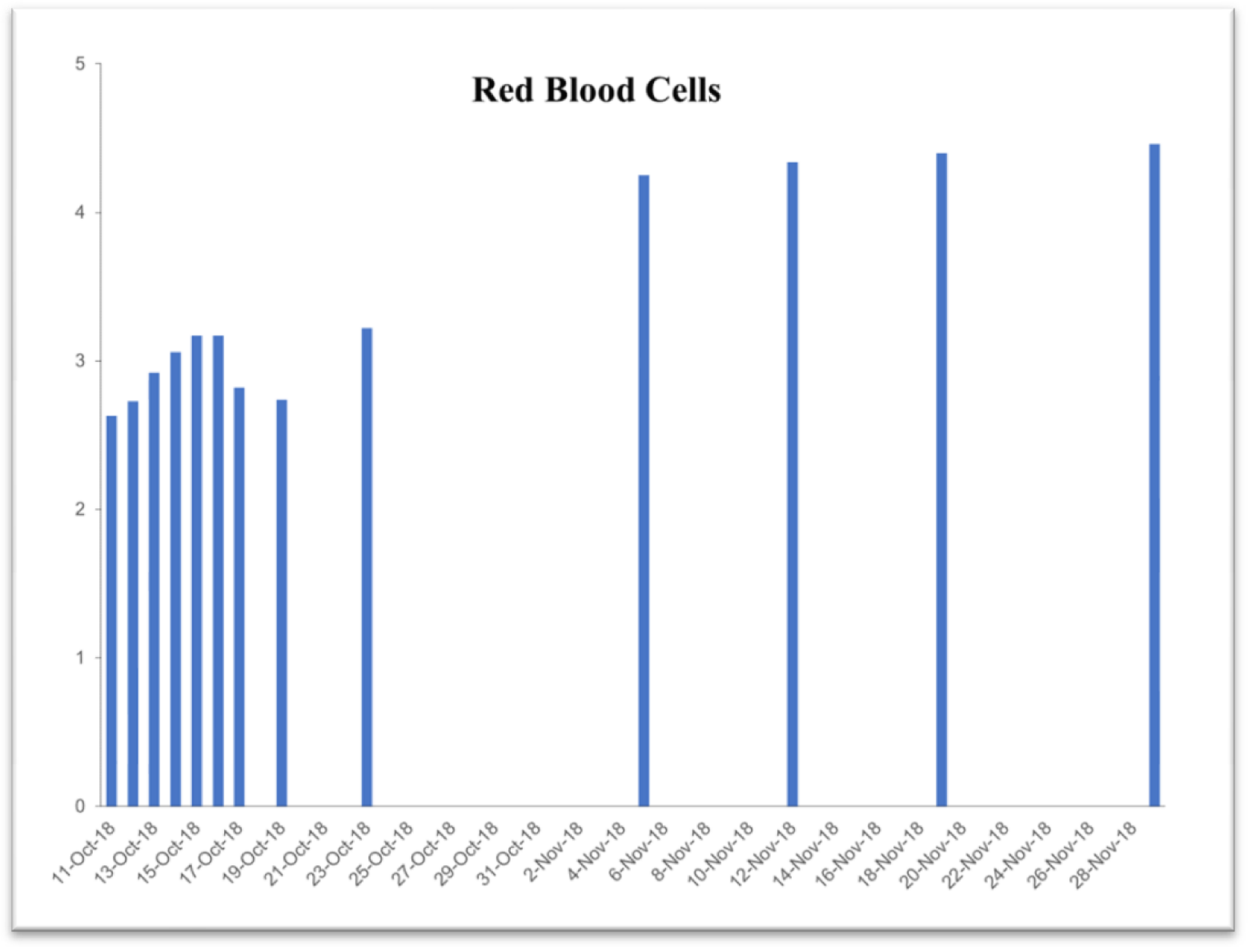
Graph represent red blood cells

**Figure 5.**
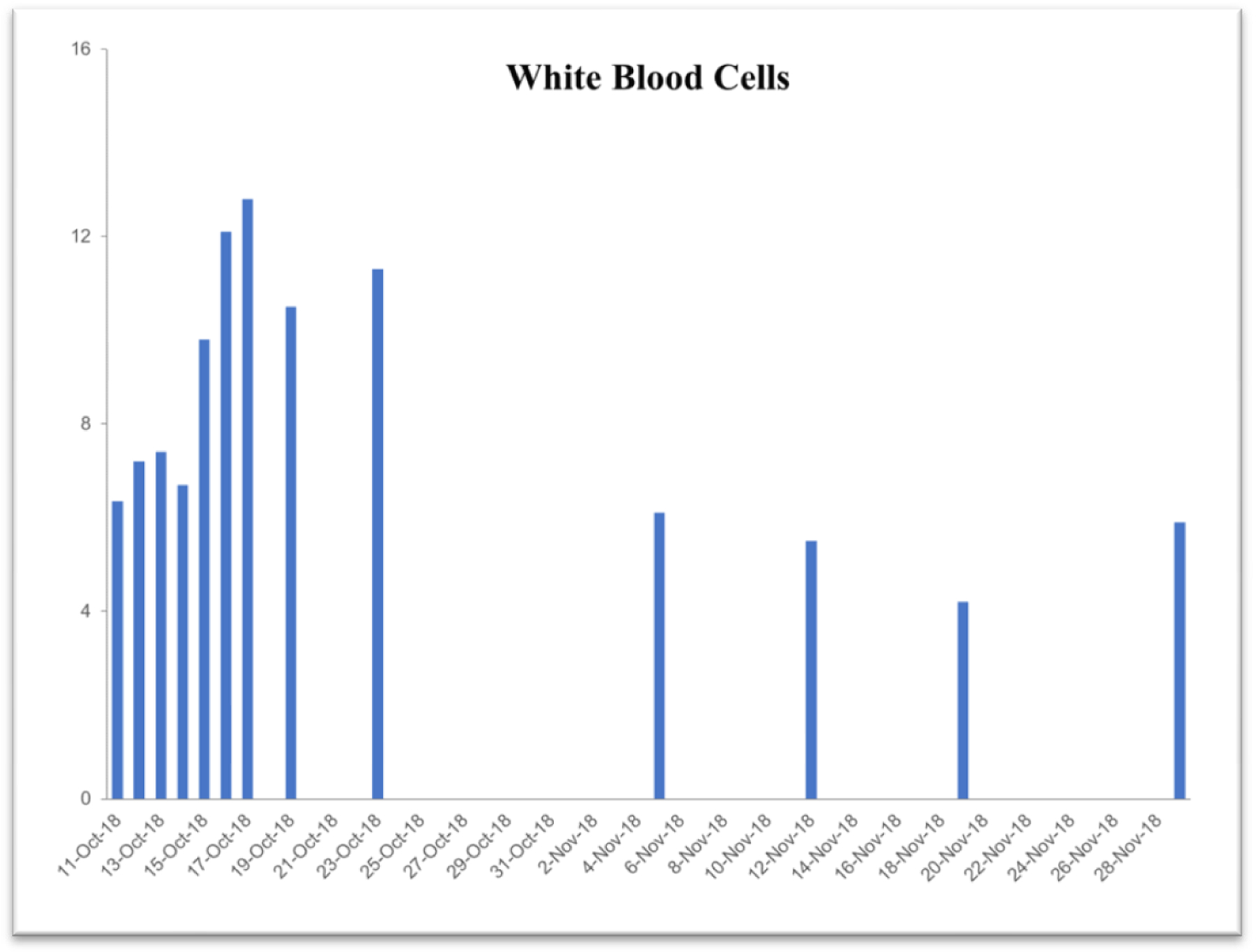
Graph represent white blood cells

This case study was performed under strict supervision of physicians. The total blood chemistry data has been summarized in Table 2, which demonstrated the benefits of the VMP35 intake. The recovery was accelerated far beyond medical and physical therapy expectations. Following daily supplementation of the VMP35, the subject regained his vitality and vigor, as well as both hemoglobin and hematocrit levels improved dramatically. Table 2 shows the evidence of significant repair to damaged blood vessels and injured tissues. In addition, platelet count became normalized. More detailed information is presented in Figures 1–5. RBCs, WBCs, Platelets, and accelerated repair of damaged tissues as evidence by the rapid recovery time and Subject’s ability to engage in winter sports activities significantly ahead of therapeutic projections.

Overall, both case studies demonstrate the beneficial effects of iron-free VMP35 in boosting immunity, hematological parameters, and accelerating competent healing, including improvements in total blood chemistry and physical health as well as a remarkable reduction in inflammatory sequela.

## Discussion

Patent-pending Prodosomed iron-free VMP35 is a liquid formulated combination of research-affirmed antioxidants, multivitamins, micronutrients, minerals, and standardized botanical phytonutrients enriched in astragalus, selected flavonoids, green tea catechins, high-potency patented pine cone extract lignans, proprietary aloe vera phytosaccharides, oligomeric proanthocyanidins and selected proprietary botanicals rich in phytochemicals, all of which have been nano-emulsified and encapsulated in the novel multi-lamellar clustoidal Prodosome^®^ absorption technology. A clinical investigation demonstrated that the iron-free VMP35 rapidly improved morphological, hematological, and rheological properties of live human blood. Moreover, this study demonstrated that the VMP35 provides adequate buffering as evidenced by the restoration of intracellular red blood cell hemoglobin within 5 minutes of intake that was sustained, and even improved, at 30 minutes post-intake. In addition, white blood cell properties, including neutrophils, significantly improved [1]. Furthermore, case study reports included in this manuscript are very encouraging and strengthen the findings of VMP35 research.

Chronic anemia syndrome (CAS) is a constellation of disorders and chronic inflammatory events caused by an increasing anaerobic/hypoxic/acidic environment, which can promote the growth of anaerobic organisms. This anaerobic environment induces a defensive expenditure of alkalinizing buffers in hemoglobin (i.e. histidine), to prevent a dangerous lowering of blood pH. In this process, iron is cleaved from heme groups and transferred out of blood circulation into other organs, like the liver, appearing to be iron deficiency anemia (IDA) and anemia of chronic disease (ACD), which has been misconceptualized. Excessive accumulation of iron in the tissues can lead to hemochromatosis, also known as ‘iron overload anemia’ (IOA).

Our research team’s evidence-based proposition is that the most effective remedy for such CAS disorders would be to replenish alkalinizing buffers (not ingesting high alkaline products) derived from appropriate nutrient resources other than iron, thereby reanimating primary cellular pH buffering capabilities and restoring healthy Hb properties, functions, and effective oxygen utilization. The benefit of this safe and routine practice would significantly reduce oxidative stress.

Based on results of the previous clinical investigation [1] on 38 subjects and case studies on the iron-free VMP35 supplement, a novel and effective treatment strategy for chronic anemic conditions, including one case of chronic anemia and one case of hemorrhagic anemia, respectively were demonstrated. The authors of this paper assert that the new diagnostic concept of chronic anemia syndrome (CAS) be established to replace the previous misconceptualized premise of iron deficient anemia (IDA) and anemia of chronic disease (ACD). Our group recognizes anemia as an etiological antecedent, a primary underlying cause to all the chronic anaerobic/hypoxic pathologies and disorders highlighted in this manuscript, including viral and bacterial pathologies.

A clinical study demonstrated that iron-free VMP35 effectively restored iron-dependent hemoglobin to RBCs and improved aerobic, morphological, hematological, and rheological properties of live human blood. Moreover, the study demonstrated that enough nutritional and phytochemical resources were available from the VMP35 to provide adequate buffering to restore intracellular RBC hemoglobin and its oxygen-carrying abilities within 5 minutes of intake that was sustained for at least 30 minutes post-intake. Properties of WBCs including neutrophils were significantly improved [1,9]. Furthermore, the physicians’ case studies are very encouraging, further strengthening the clinical findings on the VMP35.

Overall, VMP35 can induce improvements in blood properties, functionality and may serve as a novel therapeutic intervention in the restoration of hemoglobin in RBCs, neutrophils in the blood, and reverse progression of anaerobic/hypoxic pathologies, bolstering resistance to and a more resilient recovery from viral and bacterial infections. No adverse events were reported in clinical research or case studies. Further research studies are in progress.

## Conclusion

Overall, we exhibited the pioneering application of the unique “Systems Biology Approach” of VMP35 formulation, engineered using the novel proprietary Prodosome^®^ absorption technology and nano-emulsified bio-active vitamins, minerals, and phytonutrients. Results of a previous clinical study [1] were confirmed by these case studies demonstrating that the iron-free VMP35 rapidly improved iron-dependent hemoglobinization of RBCs and oxygen utilization, immune function as evidenced by improvements in neutrophils, and more rapid recovery than projected by attending physicians and other healthcare professionals. The VMP35 contains mostly botanical extracts rich in phytosaccharides, essential to form glycoproteins, which are important to integrate competent cell structures, immunity and efficient cell to cell communications [69,70]; bioflavonoids including polyphenols; and alkalinizing buffers for restoration of iron-dependent hemoglobin and cellular oxygen utilization. For example, in regard to cell to cell bonding, this type of adhesion is facilitated by bonding interactions between P-and E-selectins expressed on endothelial cells of blood vessels and the P-selectin glycoprotein ligand-1 found on microvilli ends of leukocytes [71].

Viral, bacterial/infectious, and chronic degenerative disorders are preceded by what has been previously termed iron-deficiency anemia. The authors demonstrated that this condition has been misconceptualized, and a new nosological term, Chronic Anemia Syndrome, is being proposed, owing to the cleavage of iron from heme protein to release alkalinizing histidine [1,9]. Clinical research using the iron-free VMP35 demonstrated its ability to rapidly restore iron-dependent hemoglobin in red blood cells, enhance neutrophils in the blood indicating improvement in immune activity, and induce aerobic metabolism (aka ‘aerobic glycolysis’) by enhancing oxygenated hemoglobin of circulating blood. The iron-free VMP35 improved iron-dependent hemoglobinization, cellular aerobic metabolism and immune competence in a considerable number of anemic subjects of varying ages [1,9] and as also demonstrated in two case studies cited in this paper. Furthermore, it was substantiated that nutrient repletion therapy should be a fundamental strategy to restore the healthy properties of blood, immunity, and improve strength, vitality, and physical well-being. Further studies are in progress in a larger population to reveal beneficial effects of other molecular mechanisms of action as well as possible important therapeutic applications against various viral pandemic challenges plaguing our society.

## Figures and Tables

**Table 1.** SK713 SLP encapsulating patent-pending VMP35 multivitamin, mineral & phytonutrient formula

Ingredient
Sterile R/O water
Vitamin A (Retinyl Palmitate)
Vitamin C (Ascorbic acid)
Vitamin D3 (Cholecalciferol)
Vitamin E (Alpha-tocopheryl Succinate)
Vitamin B1 (Thiamin HCl)
Vitamin B2 (Riboflavin)
Vitamin B3 (Niacin)
Vitamin B6 (Pyridoxine HCl)
Folate (from Organic Lemon Peel)
Vitamin B12 (Cyanocobalamin)
Biotin
Pantothenic acid (d-calcium pantothenate)
Calcium lactate
Iodine (potassium iodide)
Magnesium citrate
Zinc sulfate
Sodium selenite
Copper gluconate
Manganese sulfate
Chromium chloride
Potassium citrate
Choline bitartrate
Inositol
White pine, pine cone extract (Proligna^®^)
Aloe Inner Leaf Gel Concentrated 200:1 Water Extract (BiAloe^®^)

VMP35 1:1 Herbal blend:
Astragalus extract 1:1
Ginger extract 1:1
Green tea extract 1:1
Fo-ti extract 1:1
Hawthorne berry extract 1:1
Elderberry extract 1:1
Eleuthero extract 1:1
Chamomile extract 1:1
Citrus bioflavonoids (from rose hips) 1:1
Gotu kola extract 1:1
SK713 SLP (Prodosome^®^)

**Table 2.** Time-dependent improvement history of blood chemistry parameters following supplementation with the vmp35 (prodovite) since october 13, 2018. Data were taken from the penn medicine and lancaster general health laboratory

Parameters	Oct 11, 2018	Oct 23, 2018	Nov 5, 2018	Nov 12, 2018	Nov 19, 2018	Nov 29, 2018
Hematocrit (42.0–52.0%)	21.7	30.1	39.6	40.0	40.2	39.8
Hemoglobin 14–18 g/dL	7.82	9.7	12.9	12.9	13.0	13.0
Platelet Count 150–450 10*3/μl	67.5	899	443	327	332	310
RBC 4.6–6.6 10*6/μl	2.63	3.22	4.25	4.34	4.4	4.46
WBC 4.8–10.8 10*3/μl	6.35	11.3	6.1	5.5	4.2	5.9
